# Assessing the nutritional consequences of switching foraging behavior in wood bison

**DOI:** 10.1002/ece3.8298

**Published:** 2021-11-02

**Authors:** Lee J. Hecker, Mark A. Edwards, Scott E. Nielsen

**Affiliations:** ^1^ University of Alberta Edmonton Alberta Canada; ^2^ Royal Alberta Museum Edmonton Alberta Canada

**Keywords:** *Bison bison athabascae*, dietary niche, niche breadth, nutritional ecology, nutritional geometry, wood bison

## Abstract

Diet is one of the most common traits used to organize species of animals into niches. For ruminant herbivores, the breadth and uniqueness of their dietary niche are placed on a spectrum from browsers that consume woody (i.e., browse) and herbaceous (i.e., forbs) plants, to grazers with graminoid‐rich diets. However, seasonal changes in plant availability and quality can lead to switching of their dietary niche, even within species. In this study, we examined whether a population of wood bison (*Bison bison athabascae*) in northeast Alberta, Canada, seasonally switched their foraging behavior, and if so, whether this was associated with changes in nutrient acquisition. We hypothesized that bison should switch foraging behaviors from grazing in the winter when standing, dead graminoids are the only foliar plants readily available to browsing during spring and summer as nutritious and digestible foliar parts of browse and forbs become available. If bison are switching foraging strategy to maximize protein consumption, then there should be a corresponding shift in the nutritional niche. Alternatively, if bison are eating different plants, but consuming similar amounts of nutrients, then bison are switching their dietary niche to maintain a particular nutrient composition. We found wood bison were grazers in the winter and spring, but switch to a browsing during summer. However, only winter nutrient consumption of consumed plants differed significantly among seasons. Between spring and summer, bison maintained a specific nutritional composition in their diet despite compositional differences in the consumed plants. Our evidence suggests that bison are selecting plants to maintain a target macronutrient composition. We posit that herbivore's can and will switch their dietary niche to maintain a target nutrient composition.

## Graphical abstract

We assessed how changes in the types of plants herbivores influences the nutrients the animal acquires. We quantified seasonal changes in the plants consumed by wood bison (*Bison bison athabascae*) and analyzed the plants consumed for nutritional composition. Our results indicate that wood bison have a diet typical for a gazer in the winter and spring, but switch to browsing in the summer. Despite this switch the plants consumed, bison were able to maintain a consistent nutritional niche.

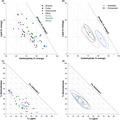

## 1 INTRODUCTION

Foraging decisions made by herbivores are motivated by the quantity and quality of available vegetation (Fryxell, 1991). How the dietary niches of herbivores respond to changes in available vegetation has been the subject of extensive research (e.g., Bailey et al., 1996; Codron et al., 2007; Illius & O’Connor, 2000; Spitzer et al., 2020). At high latitudes, seasonal fluctuations in environmental conditions influence both the quantity and quality of vegetation for herbivores (Ungerfeld et al., 2018). Functional groups (growth forms) of vegetation, such as graminoids (i.e., grasses, sedges, and other grass‐like plants), browse (i.e., woody plants), and forbs (i.e., herbaceous plants), have different nutrient compositions and their availability, quantity, and quality changes seasonally (Codron et al., 2007; Safari et al., 2011). Selection of different dietary niches should therefore correspond to consumption of different concentrations of nutrients (i.e., different nutritional niches).

Much has been done to place herbivore species along a spectrum of dietary niches based on their consumption of different functional groups of plants (e.g., Kartzinel et al., 2015; Leonard et al., 2017). Grazers have a diet dominated by graminoids, and browsers primarily consume forbs and/or browse (Clauss et al., 2010; Hofmann, 1989). Intermediate feeders have a flexible dietary niche based on resource availability, consuming intermediate levels of graminoids, forbs, and browse (Hofmann, 1989). Despite having unique dietary niches, grazers and browsers tend to have dietary niches with similar breadths (in terms of the diversity of plants consumed) that are narrower than intermediate feeders (Jung et al., 2015). The grazer/browser framework has been used to explain the coexistence or potential coexistence of multiple ruminants in a community (Abaturov et al., 2016; Fischer & Gates, 2005; Jung et al., 2015). However, many species classically defined as browsers or grazers will switch between the two foraging behaviors in response to changes in local availability and seasonal quality of vegetation. For example, two distinct populations of Sanga cattle (*Bos taurus africanus*), a quintessential grazer, had unique dietary niches with a population in a forested range browsing, while a population in grasslands was grazing (Radloff et al., 2013). Additionally, browsing elephants (*Loxodonta africana*) switch to a diet dominated by grasses and raid crops in response to new growth triggered by the onset of the wet season (Ruggerio, 1992; Vogel et al., 2020). This dietary plasticity within species and populations warrants investigation into the potential nutritional consequences of switching.

Herbivore nutritional quality is typically defined in terms of energy, concentration of limited nutrients (i.e., protein), and limiting factors such as digestibility (i.e., structural carbohydrates’ concentration), all of which influence herbivore fitness (Hamel et al., 2012; Plumb et al., 2009; van Soest, 1994). Forbs and browse tend to have greater quantities of limited nutrients than graminoids, and graminoids have more structural carbohydrates, regardless of season (Craine et al., 2010; Hecker et al., 2021). The nutritional niche of herbivores has been estimated using a multidimensional approach known as nutritional geometry (Machovsky‐Capuska et al., 2016). Nutritional geometry emphasizes the importance of nutrient balancing as a mechanism influencing foraging behavior across a range of taxa and foraging strategies (Erlenbach et al., 2014; Rothman et al., 2012; Simpson & Raubenheimer, 2012; Simpson et al., 2004). For example, disjunct populations of herbivorous mountain gorillas (*Gorilla beringei*) had different available plants, but maintained the same nutrient compositions suggesting regulation for that nutrient niche (Rothman et al., 2007). Ungulate herbivores, such as wild water buffalo (*Bubalus arnee*) and blue sheep (*Psuedois nayaur*), have a realized nutrient niche that contains high levels metabolizable energy from carbohydrates and more proteins than lipids (Aryal et al., 2015; Shrestha et al., 2020). However, the seasonal changes in the nutritional niche of herbivores and how they are influenced by corresponding changes in the dietary niche (especially in highly seasonal environments) have received little attention.

We explored how seasonal switching of herbivory behaviors relates to the composition of nutrients consumed. Specifically, we studied diets of females in a population of wood bison (*Bison bison athabascae*) herd in northern Alberta, Canada. We chose bison because they have been described as obligate grazers and have the morphophysiology of a grazer (Hofmann, 1989; Strong & Gates, 2009). However, recently, forbs and browse have been found to contribute significant proportions to some bison diets, especially during summer (Bergman et al., 2015; Craine et al., 2015; Hecker et al., 2021; Leonard et al., 2017). We elected to examine the diets of females because female bison are known to have higher quality diets that are more diverse in composition than males (Jung, 2015; Mooring et al., 2005; Popp, 1981). These differences likely arise from the additional pressures of pregnancy, parturition, and lactation that influence female diets seasonally (Berger & Cunningham, 1994; Mooring et al., 2005). We predicted that the population would have a dietary niche typical of grazers in the winter and spring when the quality of graminoids was comparable to that of forbs and browse. However, as graminoids lignify in summer, becoming less digestible, bison should select forbs and browse with higher concentrations of limited nutrients thereby switching to an intermediate or browsing dietary niche. Specifically, we consider two competing hypotheses related to switching foraging strategies: (a) Switching from grazing to browsing is a behavioral mechanism used to maximize the consumption of limited nutrients as would be evidenced by a corresponding change in the nutritional niche to include more protein, or (b) Switching from grazing to browsing is a behavioral mechanism used to maintain a particular nutrient composition as would be evidenced by a consistent nutritional niche despite changes in the plants consumed.

## 2 METHODS

### 2.1 Study system

**FIGURE 1:**
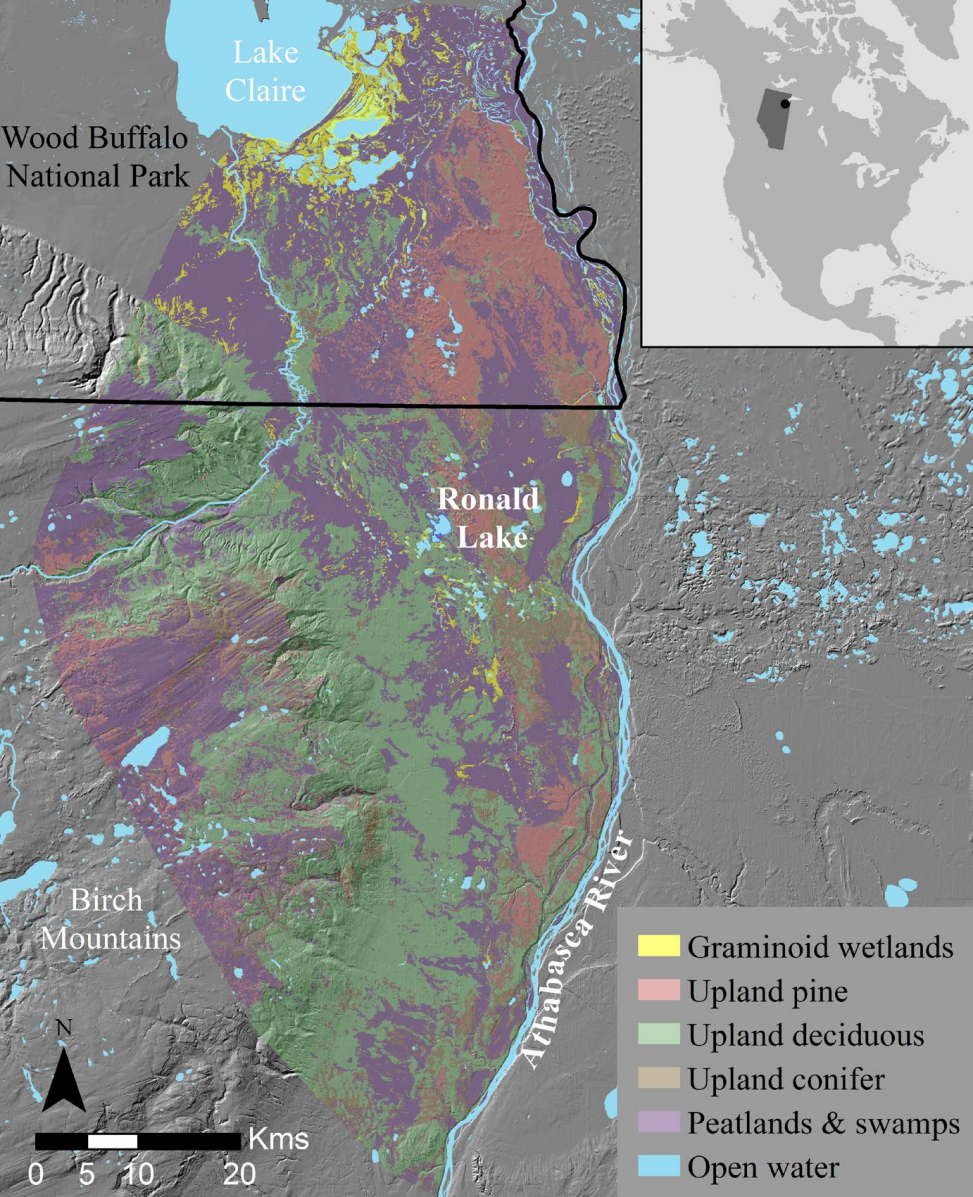
The Ronald Lake wood bison's (*Bison bison athabascae*) home range (100% minimum convex polygon) with a 15‐km buffer, cropped to the west side of the Athabasca River (DeMars et al., 2020). The colored (non‐gray) regions represent the home range and each color represents a unique land cover type. The inset map shows the study area location in Alberta, Canada

We studied females in a small population (~186 animals; Ball et al., 2016) wood bison near Ronald Lake, Alberta. The population is of significant conservation value as it has less genetic introgression with the other American bison (*Bison bison*) subspecies, plains bison (*B. b. bison*), than any other wood bison herd on record (Ball et al., 2016). Additionally, the population is free of bovine tuberculosis and brucellosis that are prevalent in nearby Wood Buffalo National Park (WBNP) populations to the north (Shury et al., 2015). The population's range extends from the southeastern corner of WBNP into Alberta's oil sands region to the south, and bordered to the east by the Athabasca River and to the west by the Birch Mountains (Figure 1, DeMars et al., 2020). The range is located in the Hay/Slave River Lowlands of the boreal forest ecoregion (Omernik & Griffith, 2014) and is composed of approximately 4% graminoid‐dominated wetlands (e.g., marshes and graminoid fens), 37% upland deciduous, 14% upland pine, 9% upland conifer, 38% peatlands and swamps (e.g., shrubby fens, bogs, swamps), and 4% open water (Figure 1, Ducks Unlimited Canada, 2016). The dominant tree species in upland habitats are quaking aspen (*Populus tremuloides*) in deciduous forests, jack pine (*Pinus banksiana*) in dry sandy sites, and white spruce (*Picea glauca*) in conifer forests (DeMars et al., 2020). Other ungulates within the range of the bison include white‐tailed deer (*Odocoileus virginianus*), moose (*Alces americanus*), and occasionally woodland caribou (*Rangifer tarandus*). Between 2013 and 2018, the government of Alberta fitted 58 females with GPS radio collars (38 Lotek Iridium Track, LOTEK wireless Inc., Newmarket, Canada; 10 Vectronics GPX Vertex Plus, Vectronics Aerospace GmbH, Berlin, Germany; and 10 Tellus GPS, FollowIT AB, Lindenberg, Sweden) with locations acquired every 90 minutes.

### 2.2 Diet content

During winter (January–March), spring (May–June), and summer (July–August) of 2018 and 2019, we visited female bison locations within 10 days of their presence to collect fecal samples that were <15 m of the GPS collar location. Samples were stored at −20°C until all samples were collected (1–8 months). To avoid overrepresenting an individual location, date, or bison, we only collected one sample per bison location in the field. Then in the lab, we randomly selected three to five fecal samples per season and extracted 5 ml of fecal matter from the center of each sample and combined these to create a composite sample. We repeated the procedure 10 times per season without replacement of fecal samples. Composite samples were then shipped to Jonah Ventures (Boulder, USA) for diet content analysis using DNA metabarcoding.

Composites were analyzed for plant DNA through sequencing of the *trnL* chloroplast introns (Craine et al., 2015). The *trnL* sequences identify plants to the species, genus, or family level. Therefore, operational taxonomic units (hereafter referred to as “taxonomic units”) are used to group sequences with 99% similarity to represent each taxonomic unit (King & Schoenecker, 2019). We used the Basic Local Assignment Search Tool (BLAST) from the National Center for Biotechnology Information to select taxonomic units by running *trnL* sequences through BLAST and selecting the taxonomic unit that had the highest percent match and was known to occur within the study area (NCBI, 2018). DNA metabarcoding reports the number of times a *trnL* sequence is read (i.e., read count) within a sample. We then calculated the relative read abundance (RRA) as the read count of a particular taxonomic unit divided by the total number of reads across all taxonomic units, which is considered a reliable proxy for diet composition (Craine et al., 2015; Deagle et al., 2019). A caveat to using RRAs to quantify herbivore diets is that they tend to overestimate plants consumed in low quantities (Deagle et al., 2019). Therefore, we used a number of selection criteria to refine out final taxonomic units: If more than one species had the same percent match with the *trnL* sequence, then we used the genera or family as the taxonomic unit; we excluded all sequences that did not occur within the study area; for the final diet content estimates, we only included taxonomic units that accounted for at least 1% of the seasonal diet. To ensure that composites were mixed evenly, we split each composite in half, analyzed each separately, and used non‐parametric Mann–Whitney–Wilcoxon tests to look for differences in RRA estimates within composite samples (Ramsey & Schafer, 2002).

### 2.3 Forage quality analysis

We collected plant samples for taxonomic units that accounted for at least 1% of the RRAs within each season. To account for potential errors associated with reducing species to taxonomic units, we collected all species that were foraged at recent bison locations. For example, the *Carex* genus was the finest taxonomic unit identified for sedges, so we collected the following three sedge species consistently foraged by bison: wheat sedge (*Carex atherodes*), beaked sedge (*C*. *utriculata*), and water sedge (*C*. *aquatilis*). At these sites, we observed how the plants had been foraged by bison and clipped the species in a similar manor (i.e., same height and same parts of the plants; Shrestha et al., 2020). Clipped samples were dried at 60°C for 24 h, then at least 26 g of each sample was cut into <5 cm pieces. Dried vegetation was shipped to Nutrilytical (Calgary, Canada) for chemical analysis of macronutrient and fiber components.

Proximate analyses were conducted on foraged plants for the macronutrient content. Standard methods from the Association of Official Agricultural Chemists were used to measure ash, moisture, crude protein, lignin, acid detergent fiber (ADF), and neutral detergent fiber (NDF), and ether extract methods of American Oil Chemists’ Society were used to calculate crude fat (AOCS, 1998; AOAC, 2006). We then determined non‐fiber carbohydrates and individual fiber components (i.e., hemicellulose and cellulose) through difference (Aryal et al., 2015; Shrestha et al., 2020):

(1) $$\text{Non - fiber carbohydrates}=100‐\left(\text{crude fat}+\text{crude protein}+\text{ash}+\text{moisture}+\text{NDF}\right)$$

(2) Hemicellulose=NDF‐ADF

(3) $$\text{Cellulose}=\text{ADF}‐\left(\text{Lignin}+\text{Ash}\right)$$

Next, we converted the percent macronutrient content to metabolizable energy values using the 4 kcal/g for carbohydrates and proteins, and 9 kcal/g for fat (Merrill & Watt, 1973). These metabolizable energy values represent the percentage of the total metabolizable energy derived from a particular macronutrient without the assistance of microbial fermentation.

### 2.4 Multidimensional nutritional niche

We evaluated changes in the seasonal diet composition of bison using nutritional geometry, a multidimensional method of assessing an animal's dietary nutrition in the context of ecological niche theory (Machovsky‐Capuska et al., 2016). We assessed the bison's dietary, macronutrient, and fiber niches. These niche estimates quantified the macronutrient compositions of plants seasonally foraged by bison thus accounting for the limitations of availability (Coogan et al., 2018). We plotted diet, macronutrient, and fiber content on right‐angled mixture triangles (RMTs); a three‐dimensional simplex, that uses the implicit *z*‐axis to geometrically display the space (i.e., niche) of three components of an animal's diet (Raubenheimer, 2011). For diet composition RMTs, we used percent content of browse (*x*‐axis), forbs (*y*‐axis), and graminoids (*z*‐axis) in the diet (Spitzer et al., 2020). In these RMTs, niches closer to the origin represent grazing behavior and niches at the *z*‐axis represent browsing. We used percent metabolizable energy for carbohydrates (*x*‐axis), lipids (*y*‐axis), and protein (*z*‐axis) to create macronutrient RMTs (Machovsky‐Capuska et al., 2016). For fiber RMTs, we used percent content of lignin (*x*‐axis), hemicellulose (*y*‐axis), and cellulose (*z*‐axis) (Aryal et al., 2015). To determine if changes in macronutrient composition were significant between seasons, we calculated the mean percent metabolizable energy of all plants consumed within each season and generated a 95% confidence ellipse around the mean (Monnette, 1990). If the 95% confidence ellipse from one season encapsulated the mean of another season, then those two seasons did not significantly differ (Fox, 2016; Monnette, 1990). We calculated confidence intervals around the means for macronutrient, fiber, and diet components to represent the nutritional components within each plant each season. We then calculated weighted means (using the RRA as the weighting factor) to represent how the components were consumed (i.e., realized niches). Finally, we calculated niche breadth and overlap for seasonal realized diet (at the taxonomic unit and forage group levels), macronutrient, and fiber niches. Niche breadths were calculated as the diversity of taxonomic units, macronutrient concentrations, and fiber concentrations while accounting for the relatedness of taxonomic units using R package *indicspecies* (De Cáceres et al., 2011; R Core Team, 2017) and compared them using Mann–Whitney–Wilcoxon tests (Ramsey & Schafer, 2002). We assessed differences in individual macronutrients and fiber components in bison diets using one‐way ANOVAs. Then, we used post hoc Tukey's HSD tests for the three seasons and four functional forage groups (Ramsey & Schafer, 2002).

## 3 RESULTS

### 3.1 Seasonal diets

We collected 129 fecal samples: 46 in winter, 38 in spring, and 45 in summer. DNA metabarcoding found 5322 unique *trnL* sequences in the female's fecal samples across all seasons, which we reduced to 119 identifiable taxonomic units accounting for 95% the herds' cumulative read count. Winter fecal samples contained 58 taxonomic units, spring samples had 66 taxonomic units, and summer samples had 57 taxonomic units. Mean read count within composite sample pairs did not differ significantly (*p* = .41). Therefore, we used the mean of the read count between pairs of composite samples to calculate RRAs.

**FIGURE 2:**
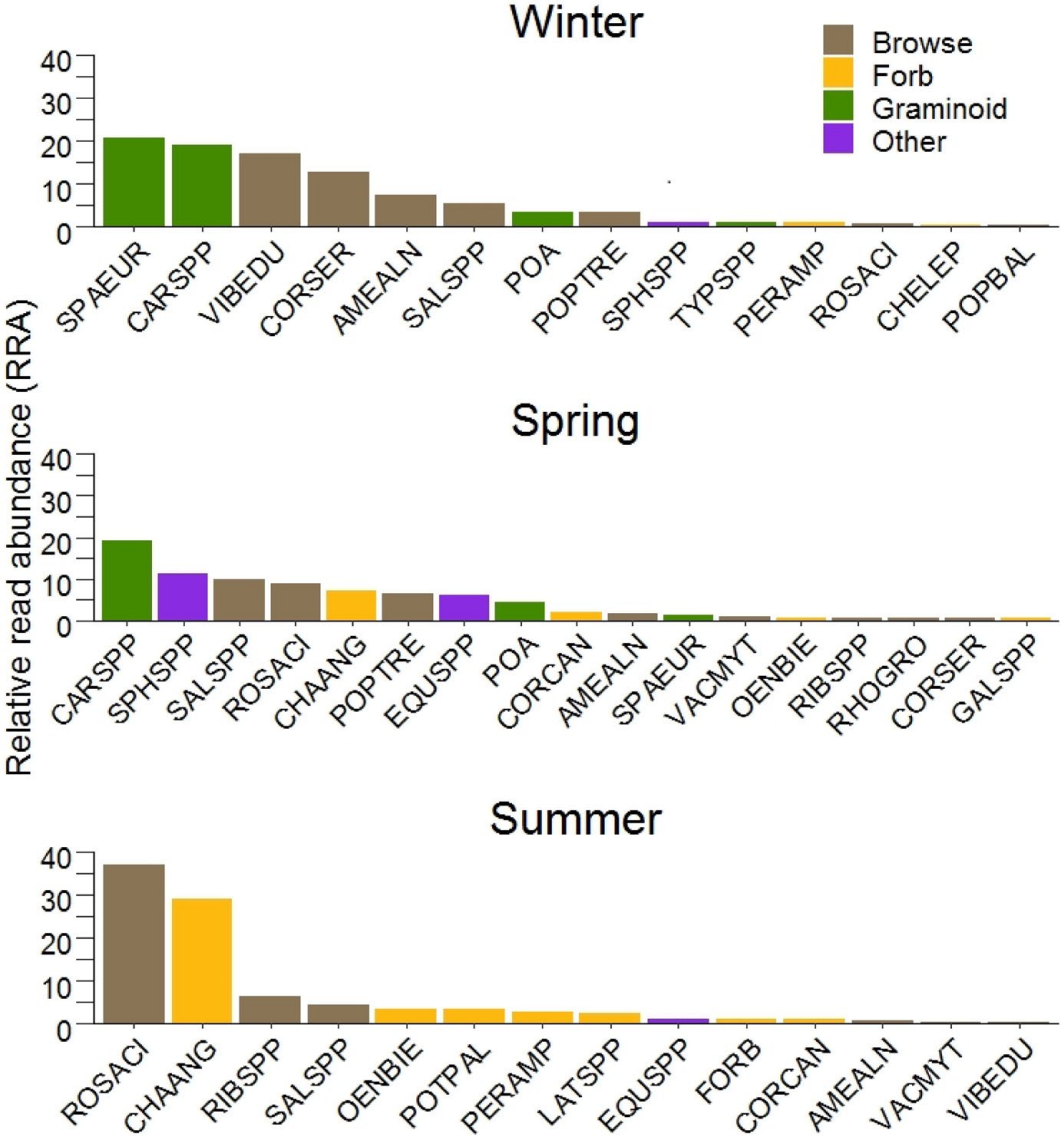
The diet composition of Ronald Lake wood bison (*Bison bison athabascae*) reported as relative read abundances (RAAs; the number of times a unique DNA sequence was found in a fecal sample divided by the total number of DNA sequences multiplied by 100) of fecal samples for three distinct seasons: spring, summer, and winter. Twenty‐five operational taxonomic units were identified overall: *Amelanchier alnifolia* (AMEALN), *Carex* spp. (CARSPP), *Chamaenerion angustifolium* (CHAANG), *Chenopodium leptophyllum* (CHELEP), *Cornus canadensis* (CORCAN), *C*. *sericea* (CORSER), *Equisetum* spp. (EQUSPP), unknown forb (FORB), *Galium* spp. (GALSPP), *Lathyrus* spp. (LATSPP), *Oenothera biennis* (OENBIE), *Persicaria amphibia* (PERAMP), Poaceae (POA), *Populus balsamifera* (POPBAL), *P*. *tremuloides* (POPTRE), *Potentilla palustris* (POTPAL), *Rhododendron groenlandicum* (RHOGRO), *Ribes* spp. (RIBSPP), *Rosa acicularis* (ROSACI), *Salix* spp. (SALSPP), *Sparganium eurycarpum* (SPAEUR), *Sphagnum* spp. (SPHSPP), *Typha* spp. (TYPSPP), *Vaccinium myrtilloides* (VACMYT), and *Viburnum edule* (VIBEDU)

Seasonal selection for different forage groups was apparent (Figure 2; Supporting information). Winter composites had 49.5% of the taxonomic units from browse, 44.4% from graminoids, 3.8% from forbs, and 1.9% from the miscellaneous plants (e.g., mosses, lichens), hereafter referred to as “other.” Two taxonomic units associated with wetland graminoids, *Sparganium eurycarpum* and *Carex* spp., had the highest RRAs of 20.6 and 19.1, respectively, followed by two browse items: *Viburnum edule* (RRA = 17.2) and *Cornus sircea* (RRA = 12.7). Spring composites contained 32.4% browse, 25.0% graminoids, 17.9% other, and 12.6% forbs. *Carex* spp. had the highest RRA (19.0) followed by *Sphagnum* spp. (RRA = 11.4), *Salix* spp. (RRA = 9.9), and *Rosa acicularis* (RRA = 9.0) in spring. Summer composites contained 51.6% browse, 44.7% forbs, 1.5% other, and 0.5% graminoids. *Rosa acicularis* was the most frequent (RRA = 37.1) followed by *Chamaenerion angustifolium* (RRA = 29.1), *Ribes* spp. (RRA = 6.3), and *Salix* spp. (RRA = 4.6).

### 3.2 Foraging behavior and dietary niches

**FIGURE 3:**
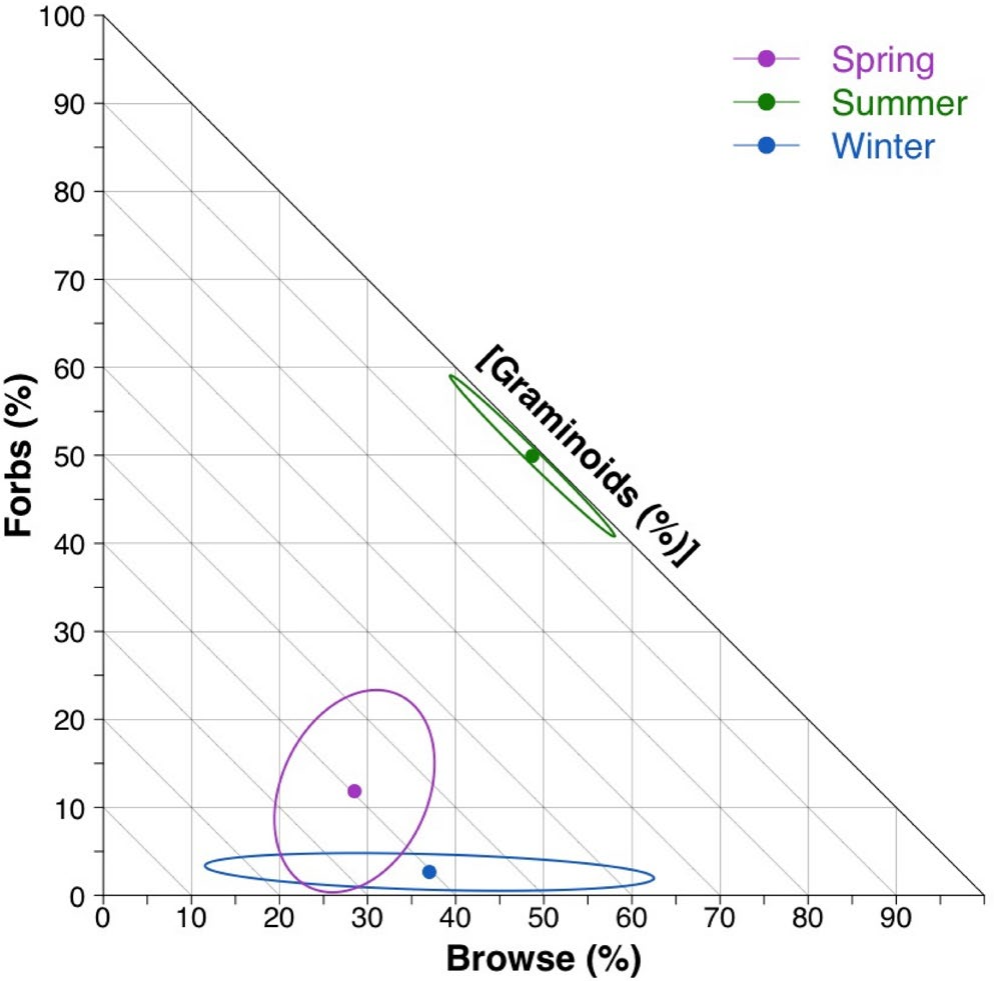
Right‐angled mixture triangle of the Ronald Lake wood bison (*Bison bison athabascae*) diet in terms of three functional forage groups: browse (woody plants), forbs (herbaceous plants), and graminoids (grass‐like plants). Each point represents the mean content of all three forage groups within each season (spring as purple, summer as green, and winter as blue) and the surrounding confidence ellipses the 95% confidence intervals. Grazers will have diets closer to the plot origin (lower left) and browsers will be closer to the *z*‐axis (right). Miscellaneous forage items such as mosses and horsetails (*Equisetum* spp.) are not considered in this plot. All other taxonomic units present in bison fecal samples are considered when calculating means and confidence ellipses

The foraging behavior during winter and spring is typical of a grazer/intermediate feeder, but in summer, the herd switched to a browsing behavior (Figure 3). At the taxonomic level, we found spring dietary niche breadth was significantly greater than summer (W = 75, *p* = .03) and winter (W = 74, *p* = .04; Table 1), but similar between summer and winter (W = 54, *p* = .40). The overlap in seasonal dietary niches at the taxonomic unit level was also significantly greater between winter/spring than summer/winter (W = 100, *p* < .01) and spring/summer overlap was significantly greater than summer/winter (W = 80, *p* = .01). The spring/summer and winter/spring overlap did not differ (W = 48, *p* = .57). At the level of forage groups, there was significantly greater niche breadth during spring than summer (W = 100, *p* < .01) and winter (W = 100, *p* < .01), but summer and winter niche breadths were similar (W = 63, *p* = .18; Table 1). Dietary niche overlap of forage groups was significantly greater between winter/spring than summer/winter (*p* = .01), but overlap between summer/winter and spring/summer (*p* = .43), and between spring/summer and winter/spring (*p* = .96) did not differ.

**TABLE 1:** Seasonal breadth and overlap of dietary (at levels of the taxonomic unit and four functional forage groups), nutritional, and fiber niches. A larger metric of niche breadth indicates greater diversity (i.e., wider niche). Similarly, a larger metric of niche overlap indicated greater overlap between the two niches in question. The taxonomic unit and forage group describe the dietary niches at the levels of the operational taxonomic units as identified by DNA metabarcoding of wood bison (*Bison bison athabascae*) fecal samples and the four functional forage groups (browse, forbs, graminoids, and other). The macronutrient and fiber niches are composed of carbohydrates, lipids, and proteins, and lignin hemicellulose and cellulose, respectively

	Breadth			Overlap		
	Winter	Spring	Summer	Winter Spring	Spring Summer	Summer Winter
Taxonomic unit	0.369	0.412	0.392	0.332	0.323	0.045
Forage group	0.205	0.329	0.251	0.773	0.613	0.549
Macronutrient	0.135	0.202	0.229	0.990	0.997	0.987
Fiber	0.309	0.319	0.315	0.995	0.976	0.981

### 3.3 Seasonal patterns in nutritional composition

**FIGURE 4:**
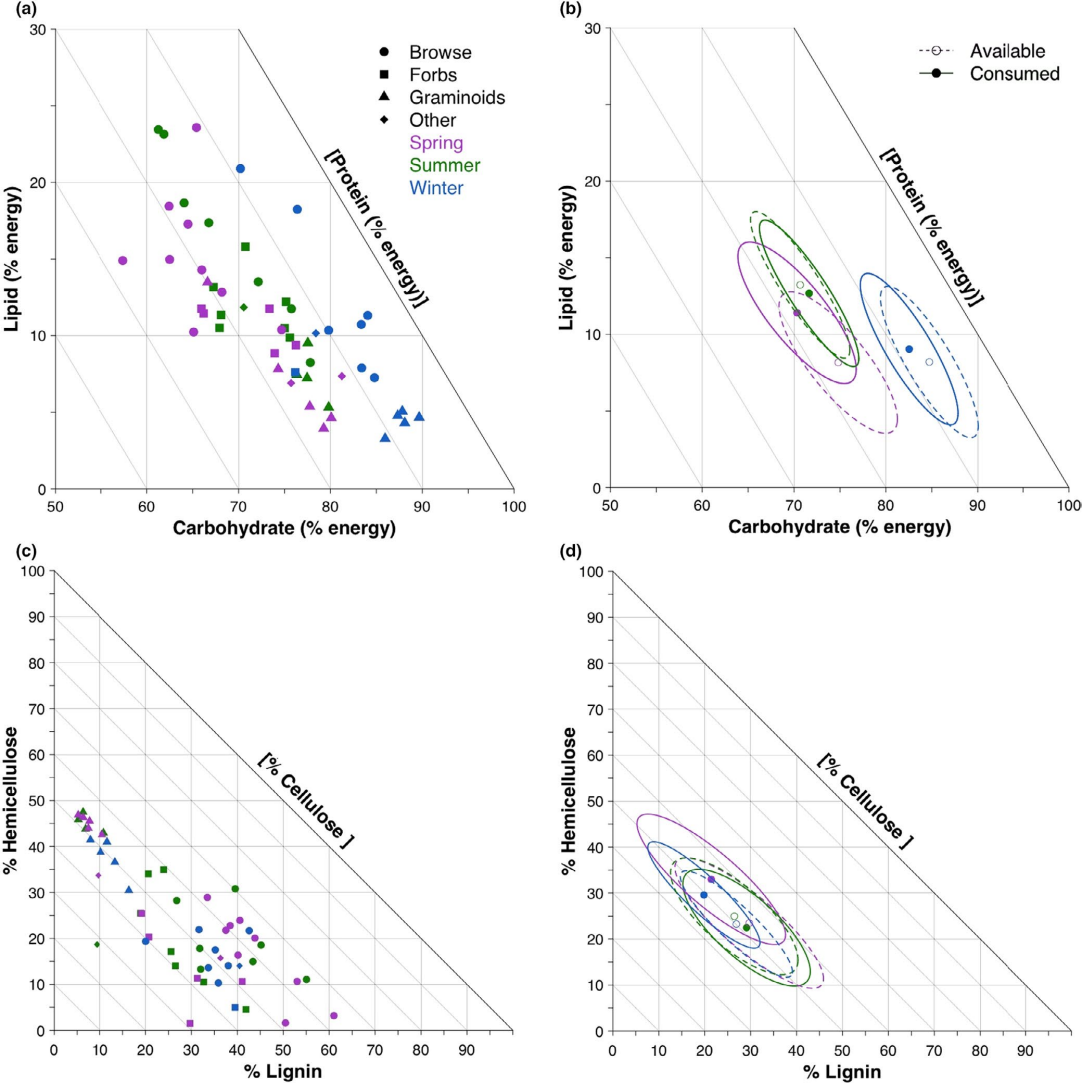
(a) Right‐angled mixture triangle (RMT) displaying the macronutrient composition of wood bison diet during three seasons: spring (May–June), summer (July–August), and winter (January–March). Symbols represent the 26 most frequently foraged plants and the functional forage group that each belongs to. (b) RMT showing the seasonal mean macronutrient composition of wood bison. The axes of these RMTs are reported as percent energy because macronutrient concentrations were converted to metabolizable energy provided by each macronutrient as described in the methods. Each point represents the mean macronutrient composition for a given season and the ellipses show the 95% confidence ellipse around that mean. Solid points and lines represent the mean macronutrient composition of the plants consumed each season weighted by their relative read abundance. Dashed lines and hollow points signify the mean macronutrient composition in the same plants if they were consumed in equal proportions. (c) RMT presenting the fiber composition of foods foraged by wood bison during the three seasons. (d) RMT depicting the 95% confidence ellipses around the mean macronutrients weighted (solid dots and lines) by the relative read abundance of each plant and the mean macronutrient content in those forages if they were consumed in equal proportions (hollow points and dashed lines)

We observed changes in nutrient compositions between some seasons, but we did not observe changes in fiber content (Figure 3; Supporting information). The digestible energy in winter diets came from 82.5% (SD = 5.6) carbohydrates, 9.0% (SD = 5.2) lipids, and 8.4% (SD = 3.0) proteins, while fiber consisted of 26.9% (SD = 12.9) lignin, 49.9% (SD = 5.8) hemicellulose, and 23.3% (SD = 12.2) cellulose. The energy from macronutrients in spring diets was derived from 70.3% (SD = 6.8) carbohydrates, 11.4% (SD = 4.9%) lipids, and 18.3% (SD = 4.1) proteins, while fiber consisted of 29.7% (SD = 17.0) lignin, 46.8% (SD = 8.9) hemicellulose, and 23.5% (SD = 14.9) cellulose. Digestible energy in macronutrients in the summer diets came from 71.6% (SD = 5.7) carbohydrates, 12.7% (SD = 5.0) lipids, and 15.7% (SD = 2.6) proteins, while summer fiber consisted of 26.4% (SD = 14.6) lignin, 48.6% (SD = 10.0) hemicellulose, and 13.4% (SD = 5.4) cellulose. The nutritional niches of winter significantly differed from spring and summer, primarily through higher carbohydrate consumption in the realized niche compared to if plants were consumed in equal proportions. However, spring and summer realized macronutrient niches did not differ despite the nutritional niches of the consumed plants being significantly different (Figure 3). Fiber components were centered around 48.2% (SD = 8.6) cellulose regardless of season and showed no differences between seasons of the consumed plants' niches and the realized niches (Figure 4). Macronutrient and fiber compositions of forage groups differed significantly within forage groups between seasons and between forage groups within each season except for browse and forbs in spring (Supporting information; Table S1).

Analysis of the consumed plants’ nutrient and fiber content showed significant changes between seasons and forage groups in the most frequently foraged plants (Table 2, Supporting information). Crude protein differed significantly between seasons with spring foods containing the most protein followed by summer, and then winter. Additionally, non‐fiber carbohydrates and ash were significantly higher in winter than summer. Between forage groups, lipids were significantly different with browse having the most lipids, followed by forbs, then graminoids and other plants. Non‐fiber carbohydrates were significantly greater in graminoids than all other groups. Browse and forbs had significantly higher moisture content than graminoids. Forbs and other items had significantly higher ash than browse. In terms of fiber, winter plants had significantly more lignin than summer foods and significantly more cellulose than spring and summer foods. Graminoids had significantly more hemicellulose content than all other functional forage groups and had significantly more dry matter than forbs or other items. Graminoids and other items contained significantly more cellulose. Lignin content was significantly higher in browse than graminoids.

**TABLE 2:** Means and standard deviations (in parentheses) of percent content for nutritional components of plants most frequently consumed by the Ronald Lake wood bison (*Bison bison athabascae*). Results of one‐way ANOVA test for differences between three seasons and four functional forage groups are reported as *p*‐values. Bold numbers are those that are significantly different and the symbol indicates the direction of the relationship. The “Carbohydrates” row refers to the non‐structural carbohydrates defined in Equation 1. All nutritional component concentrations were based on plant dry matter

	Season				Forage group				
	Winter	Spring	Summer	*p*‐value	Browse	Forb	Graminoid	Other	*p*‐value
Ash	**1.64** ^b^ **(0.33)**	1.93 (0.37)	**2.11** ^a^ **(0.37)**	<.01	**1.74** ^b^ **(0.43)**	**2.08** ^a^ **(0.20)**	1.91 (0.129)	**2.42** ^a^ **(0.70)**	<.01
Carbohydrates	**74.50** ^a^ **(5.40)**	**62.10** ^b^ **(5.15)**	**62.64** ^b^ **(5.18)**	<.01	**64.76** ^b^ **(8.57)**	**62.18** ^b^ **(3.75)**	**70.97** ^a^ **(5.12)**	**61.45** ^b^ **(7.29)**	<.01
Crude Protein	**7.55** ^b^ **(2.51)**	**16.17** ^a^ **(3.77)**	**13.72** ^c^ **(2.33)**	<.01	13.12 (5.45)	14.98 (3.22)	11.69 (4.13)	11.45 (2.08)	.25
Lipid	3.68 (2.23)	4.57 (2.20)	4.96 (2.09)	.10	**1.73** ^a^ **(0.37)**	1.43 (0.19)	**0.81** ^b^ **(0.39)**	1.15 (0.26)	<.01
Cellulose	49.85 (5.76)	46.82 (8.92)	48.60 (9.97)	.59	**42.97** ^b^ **(8.00)**	**54.88** ^a^ **(6.92)**	48.63 (2.06)	**55.51** ^a^ **(11.98)**	<.01
Hemicellulose	23.96 (12.19)	23.49 (14.94)	24.95 (13.37)	.92	**17.50** ^b^ **(7.41)**	**16.54** ^b^ **(10.91)**	**42.40** ^a^ **(4.64)**	**20.54** ^b^ **(8.98)**	<.01
Lignin	26.89 (12.86)	29.69 (17.03)	26.69 (14.61)	.77	**39.54** ^a^ **(9.32)**	**28.59** ^c^ **(8.26)**	**8.97** ^b^ **(3.28)**	**23.95** ^c^ **(16.77)**	<.01

^a^ Significantly greater.

^b^ Significantly less.

^c^ Significantly different at an intermediate level (i.e., between the greater and lesser groups).

## 4 DISCUSSION

We show that the foraging behavior of females in the Ronald Lake wood bison population changes seasonally, but bison maintained a similar macronutrient composition when possible. Niche overlap was significantly greater in the winter/spring and spring/summer than summer/winter demonstrating a gradual shift in foraging from intermediate‐grazing to browsing occurring from winter to spring and spring to summer. Spring diets of female wood bison also had a wider niche breadth compared to the narrow (i.e., specialized) niche breadth of browsing and grazing that occurred in summer and winter, respectively. Our results contribute to a growing body of evidence that suggests bison are browsers during the summer (Bergmann et al., 2015; Leonard et al., 2017; Waggoner & Hinkes, 1986). Increased forb and browse content in bison diets in spring and summer is well documented across North America (e.g., Jung et al., 2015; Larter & Gates, 1991; Schwartz & Ellis, 1981). European bison (*B*. *bonasus*), the closest extant relative of American bison, are also strict browsers in temperate forests, especially in the summer (Cromsigt et al., 2018; Kowalszyk et al., 2011; Zielke et al., 2017). DNA metabarcoding reflects where the bison acquired their protein, not biomass intake (Craine et al., 2015), and therefore is likely biased toward foods with more protein. However, studies comparing DNA metabarcoding to methods that reflect dry matter intake (i.e., microhistology) report agreement between methods (King & Schoenecker, 2019). Therefore, similar to Leonard et al. (2017), we suggest that wood bison are more of an intermediate feeder than previously thought as they exhibit a flexible foraging strategy based on availability and quality of foods. However, we caution against applying these labels to entire species, ignoring the community structure populations exist in, as the grazer/browser spectrum should only be applied within specific herbivore communities (Clauss et al., 2010; Rothman et al., 2007).

The seasonal switching of foraging behavior correlates with seasonal changes in habitat selection and annual life‐history events of bison. The Ronald Lake bison exhibit strong selection for graminoid‐rich wetlands in the spring and winter, but switch to more use of upland habitats during the summer (DeMars et al., 2020). The increased lignification and decreased protein content of graminoids between spring and summer could be a mechanism driving this switch in habitat. Additionally, in the summer, graminoid‐rich wetlands have more biting insects and less stable footing than other habitats in the herds' range, which could contribute to the selection of upland habitats (Belanger et al., 2020). For female bison, spring is the season when fat reserves are lowest as a result of a winter diet with low lipid content and catabolism of fat reserves. Spring is also the season when nutritional stress is highest due to the high energetic demands of parturition and lactation (Cunfer & Waiser, 2016; Hudson & White, 1985). We found that crude protein was in greater concentrations in consumed plants and dietary niche breadth was the greatest in spring. This suggests that bison are able to meet their nutritional targets while also consuming graminoids that contain more digestible fiber components (i.e., hemicellulose) and therefore more energy (Codron et al., 2007). During summer, female bison are putting on mass in preparation for rut, pregnancy, and winter survival. We show that they switch to a browsing strategy at this time and consume items with more non‐digestible fiber components (i.e., lignin) but also more lipids. Lastly, in winter, less protein is abundant in consumed plants. At this time, we observed a switch back to more grazing with supplemental browse consumption suggesting selection for energy‐rich foods. Interestingly, despite the switching in foraging behavior, potentially in response to seasonal energetic demands, bison were able to maintain a similar nutritional composition. Unfortunately, we were unable to obtain fecal samples during autumn and early winter (September–December). We encourage future investigations into bison diets to target this time period for dietary and nutritional ecology studies as it is poorly represented in the literature and foraging decisions during this time may influence winter survival, especially at northern latitudes.

Our investigation into the female Ronald Lake bison's seasonal macronutrient composition provides insight into herbivore nutrient availability and regulation. As herbivores, bison are restricted to a relatively narrow macronutrient niche when compared to omnivores (e.g., Senior et al., 2016) or carnivores (e.g., Tait et al., 2014). Our realized macronutrient and fiber niche measures are similar to other herbivores whose niches have been quantified, such as blue sheep (Aryal et al., 2015) and wild water buffalo (Shrestha et al., 2020). We found that the macronutrient composition of the plants most frequently foraged between spring and summer differed, but bison consumed them at frequencies that kept the macronutrient composition consistent. This suggests that bison are selecting seasonal diets for a particular macronutrient composition within the nutrient space available to them. Homeostatic regulation of macronutrient composition through the consumption of different food items has been suggested as a mechanism influencing diet selection (Simpson et al., 2004). In this study, bison consumed more lipids in the spring to maintain a macronutrient composition similar to their summer diets. The winter macronutrient niche of consumed plants did not overlap with spring or summer containing less protein and more carbohydrates. Thus, bison may not have access to enough protein to maintain their nutritional niche resulting in the realized macronutrient niche being different than spring or summer. Alternatively, targeting graminoids and carbohydrates during winter may be an adaptation to maximize short‐term energy gains in the winter when homeostatic temperature regulation put greater energetic demands on bison than other seasons (Fortin et al., 2003). Despite this difference, females had the least variation along the protein (*z*‐axis) in all three seasons suggesting that they regulated for relative protein concentration. This is not surprising as protein is more limiting to herbivores than energy (Craine et al., 2010).

Our results support our hypothesis that bison are balancing nutrients rather than maximizing a particular nutrient through their dietary switching. In a study that applied nutritional geometry to winter moose diets in Sweden, Felton et al. (2021) also showed moose maintained a particular protein to energy ratio rather than maximize consumption of either. Utilizing this multidimensional approach provides insight into how these covarying nutritional components interact and the foraging decisions animals make based on these interactions. Additionally, we used these techniques to quantify and compare multiple levels of the bison's seasonal dietary and nutritional niches: the food exploitation niche (i.e., the range of foods consumed), the food composition niche (i.e., the range of nutritional components in the foods consumed), and the realized nutritional niche (i.e., the range of nutritional components consumed; Coogan et al., 2018). It is important to note that we quantified the proportionate contribution each individual plant makes to the bison's energy supply, not the energy from fermentation and protein synthesis in the microbes. As ruminants that use foregut fermentation by the microbial community up to 80% of the total absorbable protein in the small intestine comes from microbial protein synthesis in the rumen (Storm & Ørskov, 1983; Varel & Degority, 1989). However, these microbes require readily available carbohydrates (i.e., non‐structural carbohydrates) and nitrogen is required by microbes for fiber fermentation (van Soest, 1994). Since fiber and protein are negatively correlated, the balancing of nutrients by bison may readily be interpreted as nutrient balancing to the microbes' nutritional targets. Furthermore, the diet switching behavior likely helps to maintain this nutritional composition as we have shown.

There is little doubt that bison have the morphophysiological features of a grazer (Hofmann, 1989). However, the results presented here for a population of bison inhabiting the boreal forest where forage diversity is high suggests that the cumulative macronutrient composition of the plants consumed has greater regulatory influence on bison diet selection than phenotypic traits. We did not explore other factors known to regulate herbivore foraging behavior, such as minerals (Wam et al., 2017), or plant secondary defense compounds like tannins (Windels & Hewitt, 2011). However, we do note differences in ash contents of plants between season and forage group which represents the inorganic mineral elements in plants (Hoffman & Taysom, 2005). Future investigations into the macronutrient niches of herbivores should consider the potential for macronutrient or fiber niche differences in herbivore communities classically scaled on a grazer to browser spectrum. Our work sheds light on the importance of macronutrient regulation in herbivore diet selection and we propose that this be taken into consideration when considering population viability and carry capacity analyses of herbivores.

## CONFLICT OF INTEREST

We have not published this manuscript nor is it being reviewed by any other journal.

## AUTHOR CONTRIBUTIONS

**Lee J. Hecker:** Conceptualization (lead); Data curation (lead); Formal analysis (lead); Funding acquisition (supporting); Investigation (lead); Methodology (lead); Project administration (equal); Validation (lead); Visualization (lead); Writing‐original draft (lead); Writing‐review & editing (lead). **Mark A. Edwards:** Conceptualization (supporting); Funding acquisition (lead); Investigation (supporting); Methodology (supporting); Project administration (lead); Resources (lead); Software (equal); Supervision (equal); Validation (equal); Visualization (supporting); Writing‐review & editing (supporting). **Scott E. Nielsen:** Conceptualization (supporting); Formal analysis (supporting); Funding acquisition (lead); Investigation (supporting); Methodology (supporting); Project administration (lead); Resources (lead); Software (lead); Supervision (lead); Validation (equal); Visualization (supporting); Writing‐original draft (supporting); Writing‐review & editing (equal).

## Supporting information

Figures S1 and S2

Table S1

## Data Availability

The raw diet content measure as reads of *trnl* sequences and forage quality data can both be accessed via Dryad: https://doi.org/10.5061/dryad.rr4xgxd97.
