# Supplementary material for: Assessing the nutritional consequences of switching foraging behavior in wood bison
Source: Ecol Evol. 2021 Nov 2;11(22):16165–76. doi: 10.1002/ece3.8298 (PMC8601871; doi:10.1002/ece3.8298)
Supplement: Supplementary file 1 — Figures S1 and S2 [file ECE3-11-16165-s001.pdf]

Right-angled mixture triangles of realized macronutrient and fiber niches describing differences between and withing forage groups and seasons.

Figure SI1: Mean macronutrient and fiber compositions within season. Points that lie withing another forage group or season's 95% confidence ellipse are not significantly different. (A) Annual, mean macronutrient composition of each forage group. (B-D) Mean macronutrient compositions of forage groups (graminoids, forbs, browse, other) within each season. (E) Annual, mean fiber compositions within forage group. (F-H) Shows significant within season differences based on 95% confidence ellipses among of forage groups.

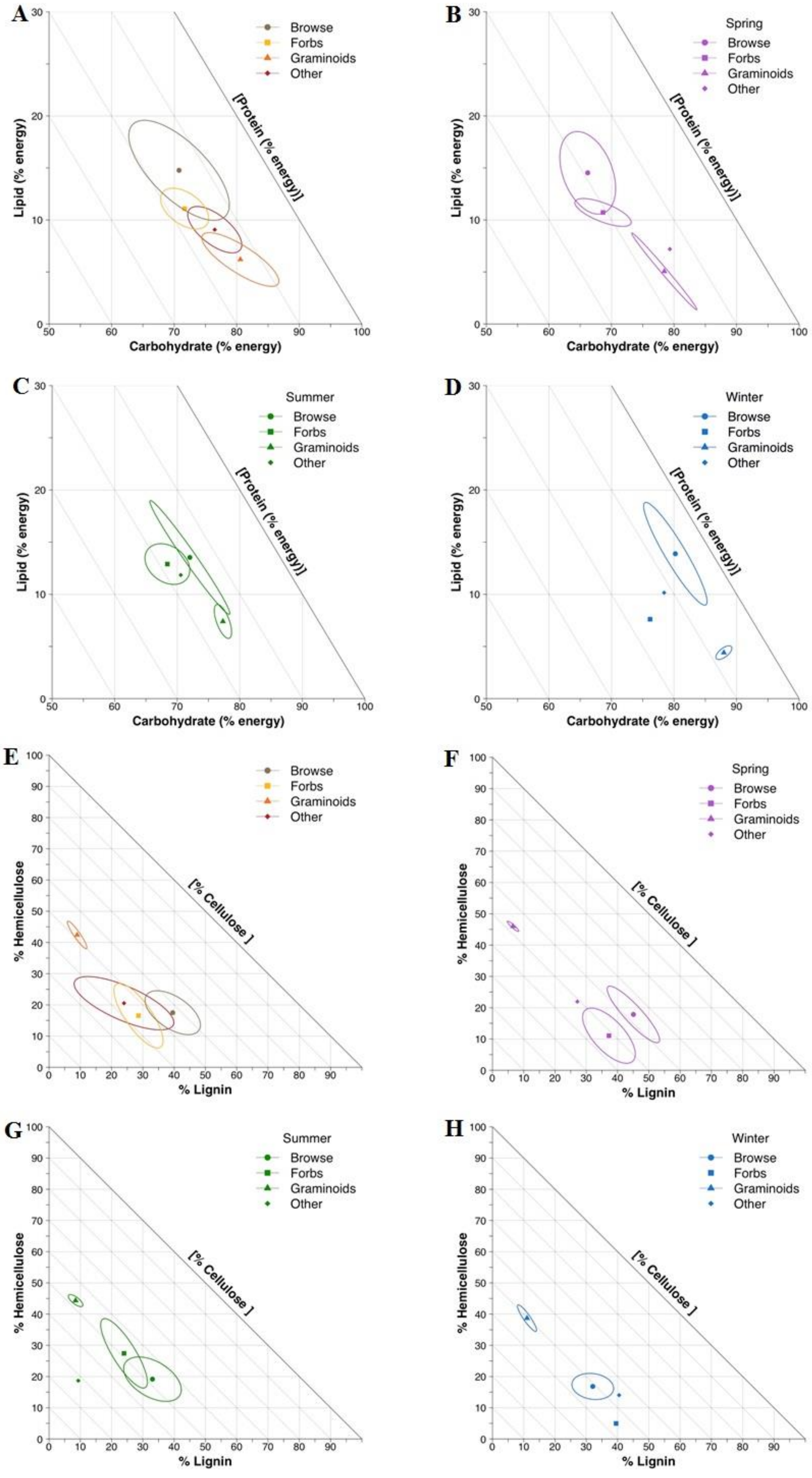

A
Browse
Forbs
Graminoids
Other
[Protein (% energy)]
Lipid (% energy)
Carbohydrate (% energy)
0
10
20
30
50
60
70
80
90
100
B
Spring
Browse
Forbs
Graminoids
Other
[Protein (% energy)]
Lipid (% energy)
Carbohydrate (% energy)
C
Summer
Browse
Forbs
Graminoids
Other
[Protein (% energy)]
Lipid (% energy)
Carbohydrate (% energy)
D
Winter
Browse
Forbs
Graminoids
Other
[Protein (% energy)]
Lipid (% energy)
Carbohydrate (% energy)
E
Browse
Forbs
Graminoids
Other
[% Cellulose]
% Hemicellulose
% Lignin
0
10
20
30
40
50
60
70
80
90
100
F
Spring
Browse
Forbs
Graminoids
Other
[% Cellulose]
% Hemicellulose
% Lignin
G
Summer
Browse
Forbs
Graminoids
Other
[% Cellulose]
% Hemicellulose
% Lignin
H
Winter
Browse
Forbs
Graminoids
Other
[% Cellulose]
% Hemicellulose
% Lignin

Figure SI2: Mean macronutrient and fiber compositions within forage groups. Points that lie withing another forage group or season's 95% confidence ellipse are not significantly different. (A-D) Mean macronutrient compositions of each season and annual mean within each forage group (graminoids, forbs, browse, other). (E -H) Shows within forage group differences based on 95% confidence ellipses among of seasons and annual means.

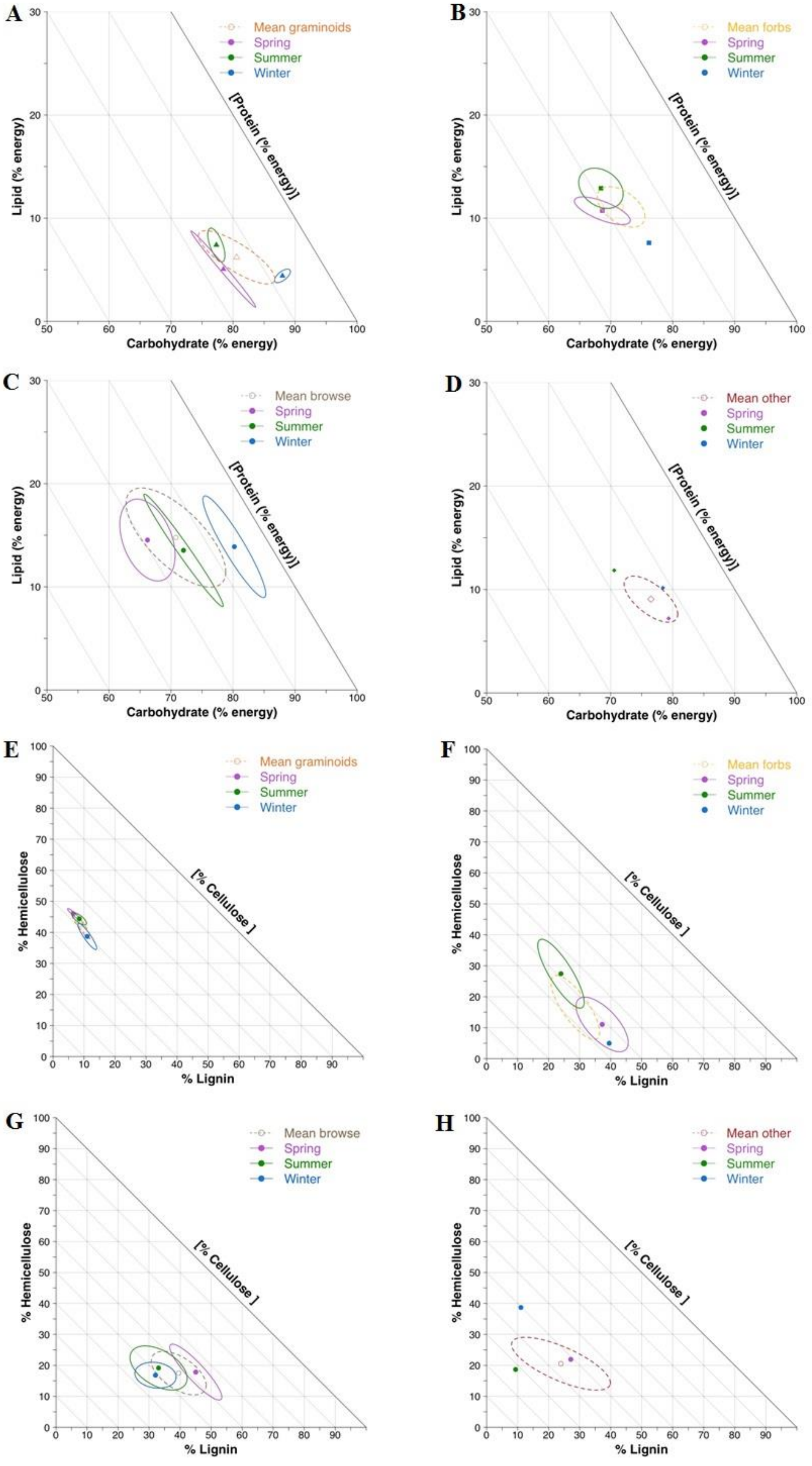
A
Mean graminoids
Spring
Summer
Winter
Lipid (% energy)
Carbohydrate (% energy)
[Protein (% energy)]
B
Mean forbs
Spring
Summer
Winter
Lipid (% energy)
Carbohydrate (% energy)
[Protein (% energy)]
C
Mean browse
Spring
Summer
Winter
Lipid (% energy)
Carbohydrate (% energy)
[Protein (% energy)]
D
Mean other
Spring
Summer
Winter
Lipid (% energy)
Carbohydrate (% energy)
[Protein (% energy)]
E
Mean graminoids
Spring
Summer
Winter
% Hemicellulose
% Lignin
[% Cellulose]
F
Mean forbs
Spring
Summer
Winter
% Hemicellulose
% Lignin
[% Cellulose]
G
Mean browse
Spring
Summer
Winter
% Hemicellulose
% Lignin
[% Cellulose]
H
Mean other
Spring
Summer
Winter
% Hemicellulose
% Lignin
[% Cellulose]
