# Supplementary material for: Assessing the nutritional consequences of switching foraging behavior in wood bison
Source: Ecol Evol. 2021 Nov 2;11(22):16165–76. doi: 10.1002/ece3.8298 (PMC8601871; doi:10.1002/ece3.8298)
Supplement: Supplementary file 2 — Table S1 [file ECE3-11-16165-s002.pdf]

Results of proximate and fiber analyses of the forage items with at least 1% relative read abundance (RRA) or that were frequently observed being consumed in the field.

Table SI1: Raw results of macronutrient and fiber analyses from items frequently foraged by the Ronald Lake wood bison herd (RLBH). The table is broken down into three seasons (winter, spring and summer) and within each season the plants are listed in order of highest to lowest relative read abundance (RRA). Proximate analyses were conducted by Nutrilytical and results presented here report moisture (water in the forage), dry matter (DM; air-dried component with all moisture removed), ash (inorganic mineral elements), crude protein (CP; total nitrogen time 6.25), soluble protein (SP; the proportion of crude protein that will be consumed by rumen microbes), and crude fat (Fat; lipids). Fiber analyses include lignin (Lign), acid detergent fiber (ADF) and neutral detergent fiber (NDF). We used results of the proximate to calculate non-fiber carbohydrates (Carbs; Equation 1) and results of fiber analyses to calculate hemicellulose (Hemi; Equation 2) and cellulose (Cell; Equation 3). Note, that three sedge (*Carex*) species each have their own macronutrient values reported, but could not be differentiated to the species level in the DNA barcoding analysis of the RLBH's diet contents. Therefore, their percent seasonal diet is reported only for the first species listed.

Winter raw values														
Species	FG	RRA	DM	Moisture	Ash	CP	SP	Fat	Carbs	Lign	ADF	NDF	Hemi	Cell
Carex aquatilis	Graminoid	-	91.4	8.6	6.7	6.6	29.0	1.7	76.5	7.2	43.4	70.9	27.5	36.2
Carex atherodes	Graminoid	-	91.2	8.8	7.1	4.9	33.0	1.8	77.4	5.7	42.4	72.4	30.0	36.7
Carex utriculata	Graminoid	19.1	91.8	8.2	5.6	7.0	32.0	1.9	77.4	8.5	43.2	73.2	30.0	34.7
Viburnum edule	Browse	17.2	93.9	6.1	5.2	5.3	26.0	8.0	75.4	20.8	47	54.7	7.7	26.2
Cornus sericea	Browse	12.7	93.4	6.6	2.3	4.5	26.0	4.9	81.8	11.8	47.5	58.9	11.4	35.7

Typha latifolia	Graminoid	11.0	93.3	6.7	5.8	9.6	28.0	1.3	76.6	11.3	48	69	21.0	36.7
Amelanchier alnifolia	Browse	7.5	93.2	6.8	4.2	5.6	25.0	4.5	78.9	18.8	48.2	55.8	7.6	29.4
Salix spp.	Browse	5.4	92.4	7.6	3.1	7.4	21.0	3.0	78.9	18.2	44.9	57.5	12.6	26.7
Poaceae	Graminoid	3.5	91.3	8.7	6.6	6.2	29.0	2.0	76.5	9.4	44.7	70.5	25.8	35.3
Populus tremuloides	Browse	3.4	87.1	12.9	6.6	8.1	18.0	8.5	63.9	22.4	41.2	52.6	11.4	18.8
Sphagnum spp.	Other	1.3	84.9	15.1	5.7	9.6	26.0	3.8	65.9	32.3	68.6	79.8	11.2	36.3
Persicaria amphibia	Forb	1.1	89.7	10.3	6.4	14.1	25.0	2.9	66.2	17.5	42.1	44.3	2.2	24.6
Rosa acicularis	Browse	0.8	90.1	9.9	4.5	7.8	18.0	3.1	74.7	17.4	43.5	48.5	5.0	26.1
Populus balsamifera	Browse	0.5	92.2	7.8	6.5	9	19.0	4.2	72.9	19.5	45.7	55.4	9.7	26.2
Winter mean (standard deviation)														
	Browse	48.6	91.5 (2.3)	8.5 (2.3)	4.8 (1.6)	7.7 (3.0)	22.3 (3.6)	4.9 (2.2)	74.1 (3.1)	18.3 (2.6)	45.0 (4.9)	53.5 (6.2)	8.5 (3.6)	26.7 (4.7)
	Forb	1.1	89.7 (0)	10.3 (0)	6.4 (0)	14.1 (0)	25.0 (0)	2.9 (0)	66.2 (0)	17.5 (0)	42.1 (0)	44.3 (0)	2.2 (0)	24.6 (0)
	Graminoid	33.6	91.8 (0.9)	8.2 (0.9)	6.3 (0.6)	6.9 (1.7)	30.2 (2.2)	1.7 (0.3)	76.9 (2.1)	8.4 (2.2)	44.3 (1.6)	71.2 (0.5)	26.9 (3.7)	35.9 (0.9)
	Other	1.3	84.9 (0)	15.1 (0)	5.7 (0)	9.6 (0)	26.0 (0)	3.8 (0)	65.9 (0)	32.3 (0)	68.6 (0)	79.8 (0)	11.2 (0)	36.3 (0)
	Total	83.5	91.1 (2.5)	8.9 (2.5)	5.4 (1.4)	7.6 (2.5)	25.4 (4.8)	3.7 (2.2)	74.5 (7.2)	15.8 (6.8)	46.5 (10.7)	61.7 (5.4)	15.2 (9.6)	30.7 (5.9)
Spring raw values														
Species	FG	RRA	DM	Moisture	Ash	CP	SP	Fat	Carbs	Lign	ADF	NDF	Hemi	Cell
Carex aquatilis	Graminoid	19.0	92.7	7.3	6.6	13.5	21.0	1.8	70.8	4.2	35.8	66.7	30.9	31.6
Carex	Graminoid	19.0	91.4	8.6	8.1	14.3	30.0	1.5	67.5	4.8	34	62.4	28.4	29.2

atherodes														
Carex utriculata	Graminoid	19.0	91.8	8.2	7.3	14.7	34.0	2.1	67.7	3.4	34.6	65.1	30.5	31.2
Sphagnum spp.	Other	11.4	88.4	11.6	6.8	9.7	30.0	2.8	69.1	29.1	67.5	80.1	12.6	38.4
Salix spp.	Browse	9.9	89.7	10.3	5.1	17.3	19.0	5.2	62.2	16.1	30.2	39.7	9.5	14.1
Rosa acicularis	Browse	9.0	89.3	10.7	6.7	17.7	20.0	5.7	59.2	11.3	22.7	29.4	6.7	11.4
Populus tremuloides	Browse	6.6	91.5	8.5	4.7	17.5	19.0	7.4	61.9	19.1	30.3	31.3	1.0	11.2
Equisetum spp.	Other	6.0	91.6	8.4	18.6	13.2	23.0	2.3	57.5	3.5	24	36.2	12.2	20.5
Chamaenerion angustifolium	Forb	5.0	85.3	14.7	6.6	18.8	38.0	4.3	55.7	10.8	23.5	26.3	2.8	12.7
Poaceae	Graminoid	4.5	92.6	7.4	5.9	16.2	41.0	3.2	67.4	4.8	36.2	64.6	28.4	31.4
Cornus canadensis	Forb	2.1	87.7	12.3	9.9	14.1	20.0	3.2	60.5	10.2	28.9	32.6	3.7	18.7
Amelanchier alnifolia	Browse	1.7	89.8	10.2	6.4	20.5	33.0	6.1	56.8	21.9	36.9	41.3	4.4	15.0
Sparganium eurycarpum	Graminoid	1.3	92	8	7.8	18.1	19.0	5.5	60.6	5.5	30.2	52.6	22.4	24.7
Ribes spp.	Browse	0.8	89.3	10.7	8.2	21.2	23.0	3.9	56.0	8.1	16.9	21.6	4.7	8.8
Vaccinium myrtilloides	Browse	0.8	87.8	12.2	3.2	13.4	5.0	4.1	67.0	10.8	22.5	26.9	4.4	11.7
Rhododendron groenlandicum	Browse	0.6	90.5	9.5	3.6	11	11.0	10.5	65.4	14.2	25.9	32.4	6.5	11.7
Cornus sericea	Browse	0.5	89.5	10.5	7.1	24.9	25.0	6.0	51.6	8.1	17.2	24.2	7.0	9.1
Populus balsamifera	Browse	0.3	92	8	6.6	18.2	15.0	7.8	59.4	15.7	30.6	31.1	0.5	14.9
Asteraceae	Forb	0.2	90.7	9.3	11.6	12.6	30.0	4.4	62.1	7.9	26.2	26.6	0.4	18.3

Lathyrus spp.	Forb	0.2	92.2	7.8	7.9	20.1	35.0	4.7	59.5	9.4	36.1	45.3	9.2	26.7
Rubus spp.	Forb	0.1	89.1	11	6.6	12.5	9.0	3.6	66.2	6.4	24.9	33.4	8.5	18.5
Spring mean (standard deviation)														
	Browse	30.2	89.9 (1.3)	10.1 (1.3)	5.7 (1.7)	18.0 (4.1)	18.9 (8.1)	6.3 (2.0)	59.9 (4.8)	13.9 (6.7)	25.9 (6.5)	30.9 (4.8)	5.0 (2.9)	12.0 (2.3)
	Forb	7.6	89.0 (2.7)	11.0 (2.7)	8.5 (2.2)	15.6 (3.6)	26.4 (11.9)	4.1 (0.6)	60.8 (1.8)	8.9 (5.0)	27.9 (7.7)	32.8 (3.9)	4.9 (3.8)	19.0 (5.0)
	Graminoid	62.8	92.1 (0.5)	7.9 (0.5)	7.1 (0.9)	15.4 (1.8)	29.0 (9.1)	2.8 (1.6)	66.8 (0.8)	4.5 (2.4)	34.2 (5.6)	62.3 (3.7)	28.1 (3.4)	29.6 (2.9)
	Other	17.4	90.0 (2.3)	10.0 (2.3)	12.7 (8.3)	11.5 (2.5)	26.5 (4.9	2.6 (0.3)	63.3 (18.1)	16.3 (30.8)	45.8 (31.0)	58.2 (8.2)	12.4 (0.3)	29.5 (12.7)
	Total	80.0	90.2 (1.9)	9.8 (1.9)	7.4 (3.2)	16.2 (3.8)	23.8 (9.6)	4.6 (2.2)	62.1 (6.7)	10.7 (10.4)	30.2 (17.0)	41.4 (5.2)	11.2 (10.4)	19.5 (8.8)
Summer raw values														
Rosa acicularis	Browse	37.1	87.3	12.7	7.4	12.4	18.0	5.2	62.4	8.4	21.7	26.4	4.7	13.3
Chamaenerion angustifolium	Forb	20.7	87.1	12.9	7.5	16.8	28.0	5.0	57.7	4.0	12.8	19.4	6.6	8.8
Ribes spp.	Browse	6.3	90.7	9.3	12.0	11.5	14.0	3.0	64.2	9.3	24.9	34.7	9.8	15.6
Salix spp.	Browse	4.6	87.6	12.4	6.8	14.2	9.0	6.9	59.7	17.3	31.2	38.3	7.1	13.9
Potentilla palustris	Forb	3.5	90.0	10.0	8.7	12.0	19.0	6.3	63.1	11.5	31.5	35.2	3.7	20.0
Vaccinium myrtilloides	Browse	3.5	87.6	12.5	3.7	11.2	2.0	4.7	67.9	14.0	24.5	35.4	10.9	10.5
Persicaria amphibia	Forb	2.7	89.1	10.9	6.6	18.9	20.0	4.1	59.5	14.7	33.5	35.1	1.6	18.8
Lathyrus spp.	Forb	2.5	93.0	7.0	8.3	18.6	36.0	4.6	61.6	9.5	37.4	50.2	12.8	27.9
Asteraceae	Forb	1.1	92.3	7.7	7.8	13.0	33.0	3.9	67.6	12.4	40.2	48.5	8.3	27.8

Equisetum spp.	Other	1.1	93.5	6.5	22.9	13.3	24.0	4.0	53.4	3.8	33.1	40.7	7.6	29.3
Cornus canadensis	Browse	1.0	87.4	12.6	11.3	10.3	5.0	4.4	61.4	6.3	17.1	26.3	9.2	10.8
Viburnum edule	Browse	1.0	89.3	10.7	11.9	13.6	15.0	9.3	54.5	17.4	34.1	40.1	6.0	16.7
Amelanchier alnifolia	Browse	0.7	90.5	9.5	7.9	14.2	15.0	9.8	58.6	23.4	37.8	42.5	4.7	14.4
Poaceae	Graminoid	0.3	92.5	7.5	5.6	14.7	37.0	3.0	69.2	7.7	40.5	71.0	30.5	32.8
Cornus sericea	Browse	0.2	88.7	11.3	9.8	15.2	21.0	7.3	56.4	6.5	17.6	20.3	2.7	11.1
Carex aquatilis	Graminoid	0.1	91.7	8.3	7.5	12.9	19.0	2.1	69.2	4.3	35.4	63.0	27.6	31.1
Carex atherodes	Graminoid	0.1	91.2	8.8	7.8	13.3	31.0	2.8	67.3	3.1	32.0	59.1	27.1	28.9
Carex utriculata	Graminoid	0.1	91.7	8.3	6.8	11.6	19.0	3.8	69.5	4.1	34.3	65.3	31.0	30.2
Galium spp.	Forb	0.1	91.7	8.3	7.3	13.0	27.0	4.2	67.3	10.0	32.4	37.7	5.3	22.4
				Summer mean (standard deviation)										
	Browse	54.4	88.6 (1.4)	11.4 (1.4)	8.9 (2.9)	12.8 (1.7)	12.4 (6.5)	6.3 (2.4)	12.8 (6.2)	26.1 (7.6)	33.0 (7.8)	60.6 (4.3)	6.9 (2.9)	13.3 (2.3)
	Forb	30.6	90.5 (2.2)	9.5 (2.2)	7.7 (0.7)	15.4 (3.1)	27.2 (6.8)	4.7 (0.9)	10.4 (3.6)	31.3 (9.6)	37.7 (11.1)	62.8 (4.0)	6.4 (3.9)	21.0 (7.1)
	Graminoid	0.6	91.8 (0.5)	8.2 (0.5)	6.9 (1.0)	13.1 (1.3)	26.5 (9.0)	2.9 (0.7)	4.8 (2.0)	35.6 (3.6)	64.6 (5.0)	68.8 (1.0)	29.1 (2.0)	30.8 (1.6)
	Other	1.1	93.5 (0)	6.5 (0)	22.9 (0)	13.3 (0)	24.0 (0)	4.0 (0)	3.8 (0)	33.1 (0)	40.7 (0)	53.4 (0)	7.6 (0)	29.3 (0)
	Total	86.7	90.2 (2.1)	9.9 (2.1)	8.8 (4.0)	13.7 (2.3)	20.6 (9.8)	5.0 (2.1)	9.9 (5.6)	30.1 (8.1)	41.5 (14.8)	62.6 (5.2)	11.4 (9.8)	20.2 (8.2)
